# Bullet-DNP Enables NMR Spectroscopy of Pyruvate and Amino Acids at Nanomolar to Low Micromolar Concentrations

**DOI:** 10.1021/acs.analchem.4c00618

**Published:** 2024-09-03

**Authors:** Pooja Narwal, Nils Lorz, Masoud Minaei, Sami Jannin, Karel Kouřil, Alvar Gossert, Benno Meier

**Affiliations:** † Institute of Biological Interfaces 4, Karlsruhe Institute of Technology, Eggenstein-Leopoldshafen 76344, Germany; ‡ Department of Biology, ETH Zurich, Zürich 8093, Switzerland; § CRMN UMR-5082, CNRS, ENS Lyon, Universite Claude Bernard Lyon 1, Villeurbanne 69100, France; ∥ Institute of Physical Chemistry, Karlsruhe Institute of Technology, Karlsruhe 76131, Germany

## Abstract

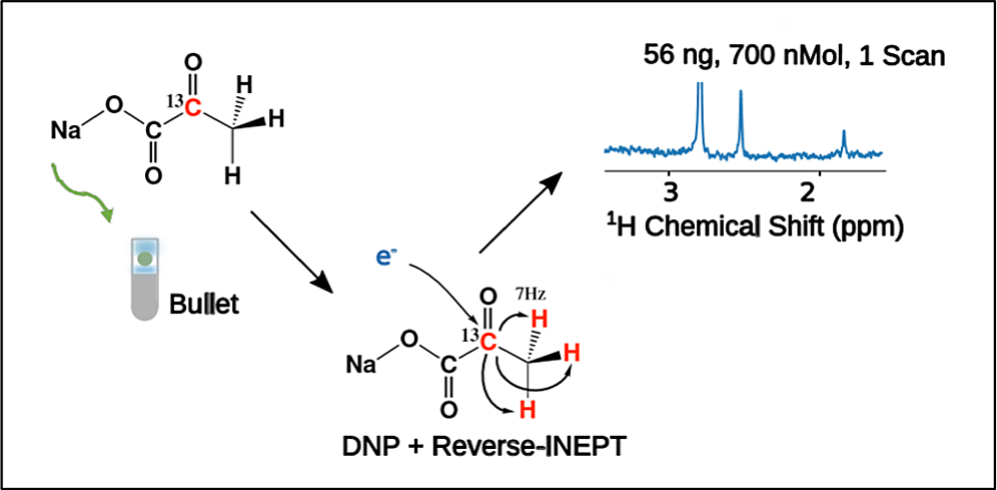

Hyperpolarized pyruvate is a widely used marker to track metabolism in vivo and a benchmark molecule for hyperpolarization methods. Here, we show how a combination of improved bullet-DNP instrumentation, an optimized sample preparation and a further sensitivity increase via a ^13^C–^1^H polarization transfer after dissolution enable the observation of pyruvate at a concentration of 250 nM immediately after dissolution. At this concentration, the experiment employs a total mass of pyruvate of only 20 ng or 180 pmol. If the concentration is increased to 45 μM, pyruvate may be detected 1 min after dissolution with a signal-to-noise ratio exceeding 50. The procedure can be extended to observe a mixture of amino acids at low micromolar concentrations.

## Introduction

Nuclear magnetic resonance (NMR) spectroscopy is a powerful probe of the structure and dynamics of living matter. The NMR signal is proportional to the nuclear spin polarization. At ambient conditions, the thermal equilibrium spin polarization amounts to only a few parts per million (ppm), a tiny fraction of its theoretical maximum of unity. Consequentially, the concentration sensitivity of NMR is limited to levels of typically 10–100 μM.^1^ The mass-sensitivity is likewise limited, although miniaturized detectors give superior mass sensitivity if the material can be investigated at high concentrations, in which case quantities as small as tens of nanograms are detectable.^2−4^

Hyperpolarization techniques can increase the nuclear spin polarization by orders of magnitude.^5^ If the target molecule can be obtained from a precursor by hydrogenation with parahydrogen,^6^ or if it can be made to bind to a suitable catalyst,^7^parahydrogen-induced polarization can boost the concentration sensitivity to the submicromolar range,^8^ and the mass-sensitivity to the picomolar range.^9^ Nanomolar concentrations of tryptophan have also been detected with so-called chemical-induced dynamic nuclear polarization,^10^ a technique that has recently been shown to work for several hundred substances.^11^

The most broadly applied hyperpolarization method is dynamic nuclear polarization. For applications to liquid-state NMR, the target molecule is mixed with a radical using a glass-forming solution. The solution is frozen, and spin polarization is transferred from the electron spins of the radicals to the nuclei. Subsequently, the frozen solid is dissolved, and NMR spectra are recorded. In dissolution-DNP, the hyperpolarized solid is dissolved inside the polarizer, using a jet of hot solvent. The solution is then transferred to a second magnet for liquid-state NMR.^12^ Dissolution-DNP can achieve polarization levels near unity, has enabled the *in vivo* tracking of human metabolism,^13^ and may provide early feedback for the treatment of human cancer.^14^ The sensitivity gains afforded by DNP and other hyperpolarization techniques may be increased further by transferring polarization from the hyperpolarized heteronucleus (typically ^13^C) to protons using a so-called INEPT sequence.^15−17^

The high polarization levels attainable with D-DNP, however, do not translate into a corresponding boost in mass-sensitivity. Several milliliters of solvent are used to prevent freezing during the dissolution process, leading to excessive dilution for mass-limited samples. Attempts to apply DNP to mass-limited samples using an immiscible heat-carrying cosolvent^18^ or in situ detection with a rapid-melt step inside the polarizing device^19,20^ have thus far not resulted in high-resolution liquid-state spectra.

We recently introduced a variant of the D-DNP experiment named bullet-DNP. In bullet-DNP, the hyperpolarized material is rapidly transferred to the liquid-state NMR magnet as a solid, and dissolved in aqueous solution inside a solvent reservoir upon arrival. The solvent reservoir is pressurized, and the flow of the solution is controlled via a pinch valve, located between the solvent reservoir and the NMR tube.^21^ Since the dissolution is carried out in a warm environment, the solution volume can be chosen to match the volume of the NMR detector and superior mass sensitivity can thus be achieved.

Here, we show that bullet-DNP can be used to detect pyruvate, a benchmark molecule for hyperpolarization, in a single scan at concentrations down to 250 nM, using sample masses as low as 20 ng. The detection is made possible through improvements to the bullet-DNP instrument, an optimal DNP host material and sample preparation, and a reverse INEPT sequence that transfers carbon polarization to protons for increased sensitivity during detection.

## Results and Discussion

**Figure 1:**
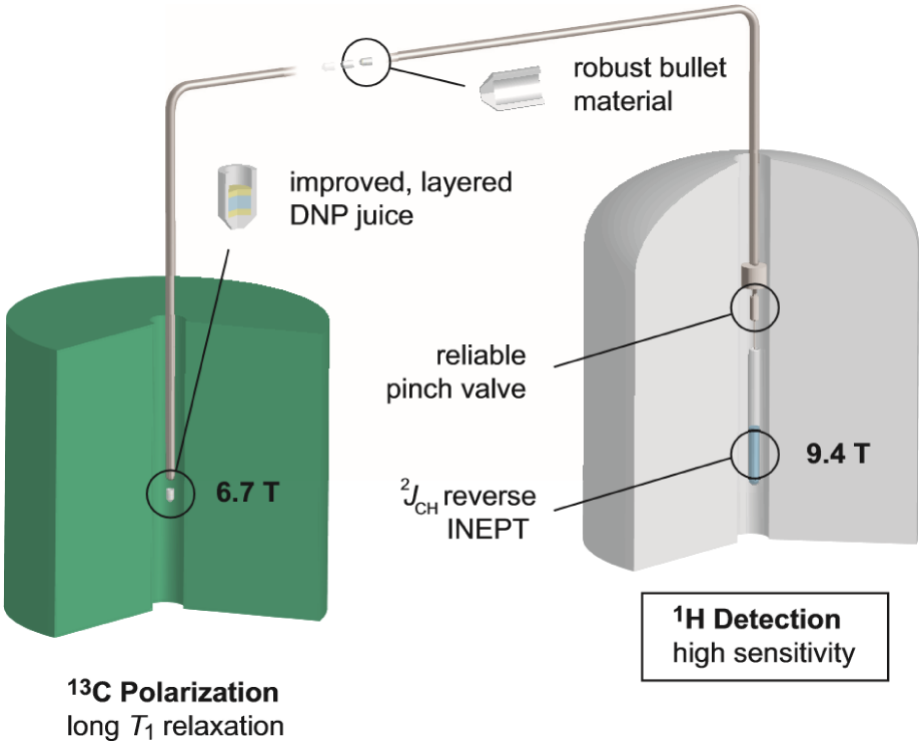
High sensitivity presented in this work is achieved by a combination of innovations concerning the bullet DNP instrument (circles) and the general experimental approach (boxes). The improvements to the DNP instrument (robust bullet container material, novel bullet content composition, redesigned pinch valve) lead to higher repeatibility and in turn to increased resolution and higher signal intensity. The general experimental approach exploits the long T_1_ of carbon in position 2 of pyruvate to limit magnetization losses during transfer and dissolution, and the subsequent reverse INEPT increases sensitivity by detection on ^1^H.

The improvements to the DNP instrument presented here concern resolution and repeatability and are summarized in Figure 1. Improving resolution compresses the signal into a smaller frequency band, thereby increasing sensitivity. Small molecules exhibit slow transverse relaxation and can be detected with the highest sensitivity if the magnetic field across the detection volume is homogeneous down to the level of 1 part per billion. Such a homogeneity is routinely achieved under static conditions by adjusting currents in the shim coils inside the NMR magnet. However, it is difficult to achieve the same resolution in DNP experiments with aqueous solutions due to degassing of the solution, and changing shim requirements upon removal and reinsertion of the injection device. Degassing may be suppressed through the application of back-pressure.^22,23^ For optimal shims, it is then desirable to leave the injection device inside the NMR magnet between experiments. To this end, the injection device needs to be automated, which includes reliable ejection of empty bullets.

**Figure 2:**
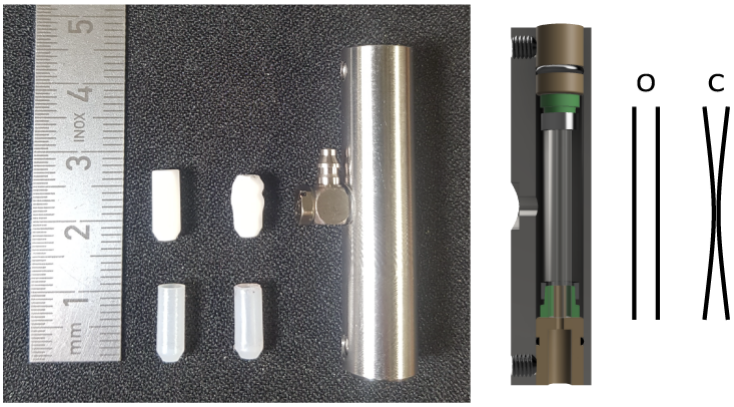
Bullets made from polyethylene (bottom) and PTFE (top) before (left) and after (right) the shot. The polyethylene bullets show signs of wear but no structural damage and hence can be removed from the injection device without removing the latter from the magnet. On the right, we show the pinch vale, along with a computer image of its cross-section, and a sketch of the silicon tubing in the open (O) and closed (C) state. The titanium valve body houses a silicon tube that is connected to the top and bottom valve ports using 1/8” compression ferrules. The PEEK valve ports accept commercially available IDEX TinyTight 1/16” microfluidic connectors. O-rings on the valve ports ensure that the valve body is leak-tight, and grub screws are used to hold the ports in position. The valve is closed by applying pressurized air to the pinch valve body via the elbow connector (SMC *M*-3ALU-2) on the left. The application of pressure pinches the silicon tubing as indicated on the right, closing the valve.

In bullet-DNP experiments, the abrupt stop of the bullet above the solvent reservoir leads to damages to the bullet, and the acculumation of bullet fragments inside the injection system. In our previous work^21,24^ we used bullets made from PTFE (Teflon). In comparison to other plastics such as PEEK, Kel-F and VESPEL, PTFE remains ductile at low temperatures, yet bullet fragments stil caused blockages of the injection system, and frequently the bullet broke into large fragments, calling for removal and manual cleaning of the injection device.^24^ It turns out that ultrahigh molecular weight polyethylene (UHMW-PE) is broadly superior for the transfer of hyperpolarized solids. Indeed, UHMW-PE exhibits a 6-fold higher impact strength than PTFE.^25,26^ With UHMW-PE bullets (hereafter PE-bullets), the formation of chips is substantially reduced, and, over more than one hundred shots, we have not observed a single complete breakdown of any bullet. A picture of a PTFE and a PE-bullet before and after the shot is shown in Figure 2. The PTFE bullet is damaged to an extent that an automatic removal from the injection device is not feasible anymore. By comparison, the PE-bullet shows only minor signs of wear. While not all PTFE bullets show the deformation shown in Figure 2, the PE-bullets, due to their superior impact strength, never deform to an extent that would necessitate manual removal of the bullet from the injection device and therefore removal of the injection device from the magnet.

A key component of the injection device is the pinch valve, which controls the flow of liquid from the solvent reservoir into the NMR tube. The valve has to be nonmagnetic, fit inside the bore of the magnet with an axial geometry, and withstand the back-pressure (3 bar in our experiments). Such valves are not available commercially. Previously, we constructed a pinch valve from a 1/8” Swagelok union. The compression ferrules of the union were used to compress 1/8” OD flexible silicon tubing onto 1/16” PEEK tubing, and a port was added on the side of the union to allow pressurization of the silicon tubing. The valve body was sealed by tightening the nuts of the union, but this process exerts torque onto the silicon tubing, which causes the tubing to twist to an unpredictable degree.

An improved valve design is shown in Figure 2 (right panel). Here, the ports of the valve are machined from PEEK and accept standardized microfluidic connectors. Toward the inside, the ports have an OD of 1/16″, and the silicon tube is fixed to the ports by pressing compression ferrules toward the port using a small aluminum clamp. Then, the silicon tube is inserted into a titanium body, and secured in position with grub screws. O-rings on the ports seal the valve body when it is pressurized via a port at the side. No torque is exerted on the silicon tubing, and the flow characteristics of the valve are stable.

With the new bullet material and the new pinch valve, the injection device achieves a residence time inside the NMR magnet of typically 2 weeks, during which we can routinely record ^1^H spectra with a resolution of up to 4 Hz in aqueous solution. It may be possible to increase the resolution further by optimizing the positioning of the NMR tube assembly and its internal capillary.

**Figure 3:**
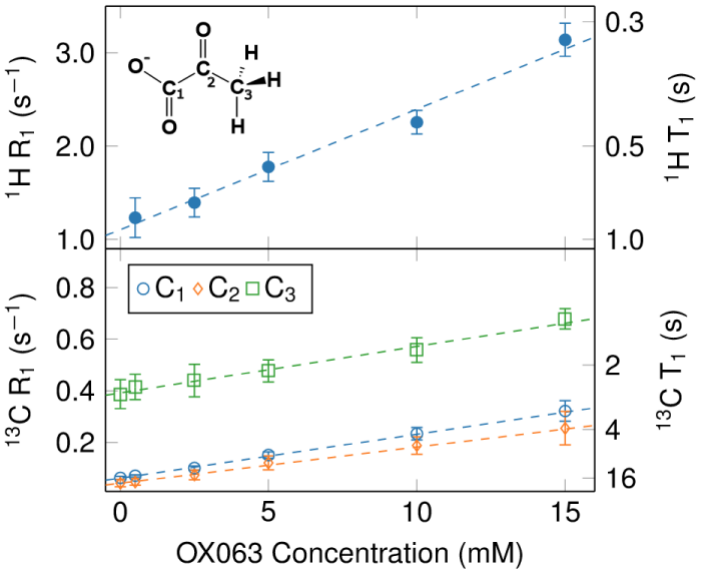
Spin–lattice relaxation rates for pyruvate vs concentration of OX063 radical, measured at a magnetic field of 9.4 T in tris-buffer at pH 7.2. While the ^1^H relaxation rate exceeds 1/s and increases rapidly with increasing radical concentration, the carbon relaxation rates are smaller than 0.1/s. The dotted lines are linear fits to the data, with fit parameters given in the Supporting Information.

The sensitivity of the experiment may be boosted further by detecting ^1^H instead of ^13^C. Fundamentally, a higher signal-to-noise ratio (SNR) is achieved by inductive detection of NMR signals at the 4-fold higher frequency of ^1^H. However, *R*_1_ of ^1^H is faster than 1/s, especially in the required presence of radicals (Figure 3), such that hyperpolarization on ^1^H would be quickly lost during the transfer and solvation process. For the highest sensitivity, we thus choose to hyperpolarize ^13^C nuclei in the DNP step, and transfer their polarization to protons using a reverse INEPT sequence.

The most efficient strategy to polarize low-abundance, low-γ nuclei is to hyperpolarize high-γ nuclei such as protons and transfer their polarization to the low-γ-nuclei using Hartmann–Hahn crosspolarization^27^ or an adiabatic transfer.^28,29^ However, such a strategy typically requires the use of a broadband radical such as 4Hydroxy-TEMPO (TEMPOL), which compared to a narrow-band radical like trityl OX063 causes fast relaxation during the solid-state transfer of the hyperpolarized sample.^30,31^ Here, we therefore chose to use the trityl radical OX063, and polarize ^13^C nuclei directly. As can be seen by comparing our data to published data on TEMPOL relaxivity,^32^ trityl also causes much less liquid-state relaxation than TEMPOL.

**Figure 4:**
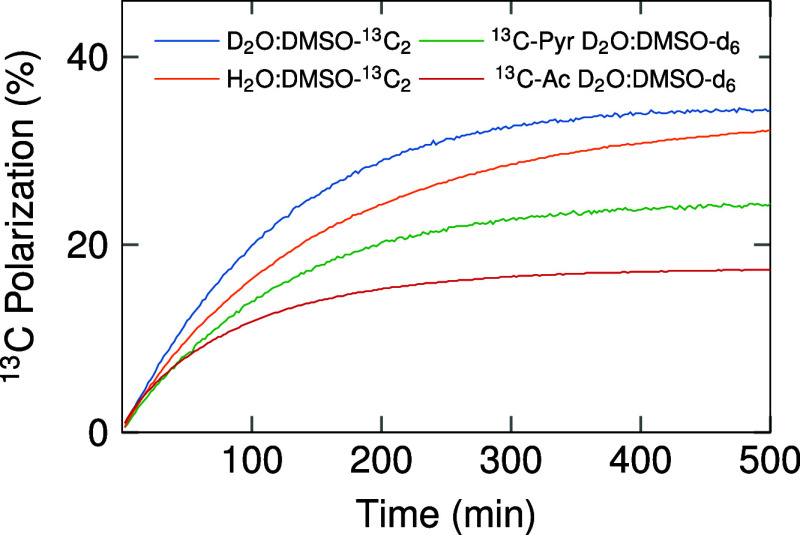
DNP buildup curves for 2-^13^C pyruvate at 6.7 T with 15 mM OX063 trityl radical at a temperature of 1.5 K. The green curve shows a buildup of 1 M 2-^13^C Pyruvate in D_2_O:DMSO-*d*_6_ (2:1). The red curve shows a buildup of 84 mM 2-^13^C pyruvate with 1.6 M 1-^13^C acetate in D_2_O:DMSO-*d*_6_ (2:1). The blue and orange curves show buildups of 100 μM 2-^13^C pyruvate in D_2_O:DMSO-^13^C_2_ (2:1) and H_2_O:DMSO-^13^C_2_ (2:1), respectively. For these two buildups, 1 M of DMSO is substituted with doubly labeled DMSO-^13^C_2_. The polarization is calculated by comparing the signal intensity to a thermal equilibrium signal of a pyruvic acid sample, normalizing for sample volume and molarity for each measurement.

Direct ^13^C-DNP requires carbon–carbon spin diffusion, and hence, a sufficient concentration of ^13^C in the sample. We therefore conducted DNP buildup experiments for various sample formulations. The results, shown in Figure 4, reveal a strong dependence of the attainable polarization level on the diffusion agent, as well as a dependence of the buildup dynamics on sample deuteration.

In order to achieve a high polarization, we experimented with various combinations of solvents and different concentrations of diffusion agents. First, we directly polarized a 1 M solution of 2-^13^C Na-pyruvate in D_2_O:DMSO-d6 (2:1). After 6 h of buildup, we obtained a polarization of approximately 25% (green curve in Figure 4). However, applying this approach at the nanogram scale results in picolitre sample sizes, which cannot be handled with the required precision. We therefore decided to work with a low pyruvate concentration, but make use of a ^13^C labeled spin diffusion agent in order to facilitate spin-diffusion. We initially used 1-^13^C Na-acetate but the resulting solid state polarization was only 15% (red curve in Figure 4). The buildup curve shows a fast buildup for short times, which may be linked to a partial aggregation of the acetate in the solution. Using Na-acetate, we also observed low pyruvate polarization levels for pyruvate concentration below 100 μM in the solid, indicating that the final pyruvate polarization is due to interpyruvate spin diffusion, and that Na-acetate is not a good spin diffusion agent in D_2_O:DMSO-d6. We provisionally attribute this finding to an uneven distribution of acetate in the frozen solid. In order to obtain an even distribution of the diffusion agent, we explored the use of ^13^C labeled DMSO solvent as a spin diffusion agent, at a concentration of 1 M. This compositional change greatly enhanced spin diffusion, resulting in a high ^13^C polarization of 35% (blue curve). Additionally, we attempted substituting H_2_O for D_2_O to observe how protonation affected the carbon polarization. Since trityl does not drive proton hyperpolarization, this would reduce the heat load onto the hyperpolarization process, possibly resulting in higher polarization levels. The resulting buildup (orange curve in Figure 4) indicates that this strategy may indeed lead to higher polarization levels, but the buildup is substantially slower than for the deuterated sample, and after a polarization time of 8 h, the polarization level of the protonated sample is still lower than that of the deuterated sample. We provisionally attribute this effect to strong static proton-carbon dipolar couplings that may hamper carbon–carbon spin diffusion. This is to be contrasted with the role of protons in magic angle spinning (MAS) NMR, where averaged, and thus weaker, proton carbon dipolar interactions facilitate spin diffusion by providing the energy that is released or absorded in a carbon–carbon flip flop of two carbon resonances that have, e.g., a different chemical shift.^33,34^ In magic angle spinning experiments, averaged proton dipolar couplings thus promote spin diffusion, whereas they appear to hamper spin diffusion in the experiments reported here. Figure S4 shows that a repetition of the buildup experiments gives similar results, hinting toward their good repeatability. All polarization levels were estimated by comparing the integrated spectral intensities to the thermal equilibrium signal of a 30 μL sample of 1-^13^C-pyruvic acid with 15 mM trityl at 1.6 K, and normalizing for the abundance of ^13^C in the different samples.

In order to explore the limits of the setup for highest mass sensitivity, we aimed at detecting pyruvate at submicromolar concentrations in a single acquisition. We decided to work with small beads (1.5 to 5 μL) of hyperpolarized material to limit the concentration of diffusion agent in the subsequent liquid-state NMR experiment. For a reliable ejection of the hyperpolarized material upon arrival of the bullet, a 4 μL bumper layer was first inserted into the bullet and frozen in liquid nitrogen. Then 1.5 to 5 μL of the pyruvate sample were pipetted into liquid nitrogen, and the resulting bead was inserted into the bullet using tweezers. A third layer of 10 μL water:glycerol (50:50) was added on top to seal the sample and limit sample heating during transfer. Care was taken to prevent mixing of the layers and for some experiments we suppressed mixing by placing a frozen water:glycerol bead between the pyruvate sample and the final layer.

The bullet was inserted into the polarizer, and hyperpolarized for approximately 6 h at 6.7 T, with the long polarization time being due to the low abundance of ^13^C in the sample. The polarization buildup was monitored by observing the solid-state ^13^C signal, which is dominated by the contribution from DMSO-^13^C. After completion of the buildup, the bullet was ejected automatically from the polarizer and shot into an injection device inside the 9.4 T liquid-state NMR magnet. The bullet’s payload was dissolved in 700 μL of buffer, and following a delay of 2 s, the solution was pushed into the NMR tube assembly, and back pressure was applied.

**Figure 5:**
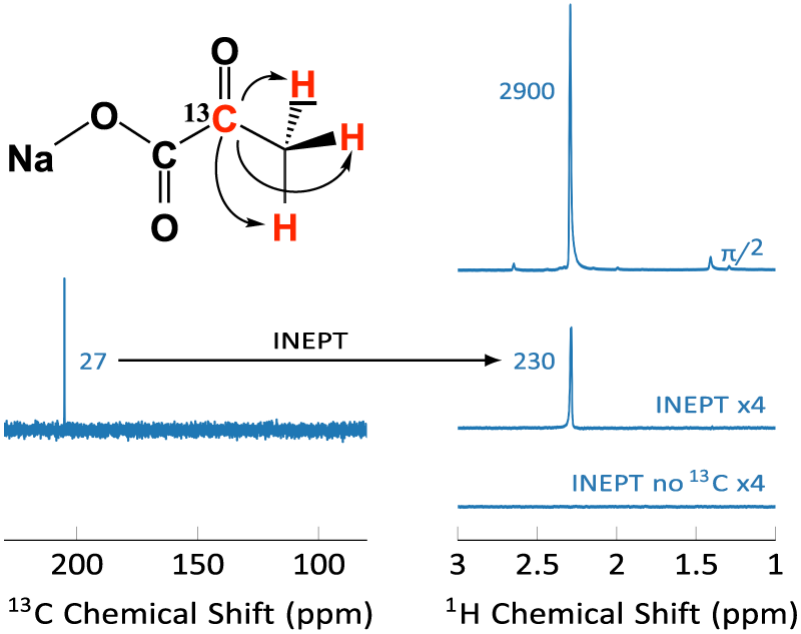
Sensitivity comparison of direct carbon detection and carbon detection via ^13^C–^1^H reverse INEPT for 2-^13^C-pyruvate. (a) The ^13^C spectrum exhibits a signal-to-noise of 27. (b) For a direct acquisition (top), the proton spectrum exhibits an SNR of 2900, whereas the INEPT based sequence (middle) yields an SNR of 230, i.e., an approximately 12-fold reduction, but an approximately 8-fold increase over the ^13^C SNR. The signal indeed originates from the ^13^C magnetization, and vanishes if the carbon RF power is set to 0 (bottom). Eight scans were averaged for each trace, the receiver gain was set to its maximum value for all data sets, and an exponential line broadening of 1 Hz was applied. Further details are given in the Supporting Information.

Instead of detecting the hyperpolarized ^13^C signal directly, the sensitivity of the NMR detection was increased further using a reverse INEPT sequence. The SNR improvement that may be obtained with INEPT, compared to direct ^13^C detection, is shown in Figure 5.

In thermal equilibrium, the highest sensitivity is obtained by detecting the ^1^H signal, with an SNR of 2900. The experiments described here detect the more long-lived ^13^C polarization. Starting from thermal equilibrium carbon polarization at 9.4 T, the ^13^C spectrum exhibits an SNR of 27. By comparison, a reverse INEPT experiment, employing the carbon thermal equilibrium polarization as its starting point, yields an SNR of 230, corresponding to an 8-fold improvement. The attained SNR via INEPT is very close to the theoretical estimate,^16^ SpinDynamica^35^ simulations and details of the pulse sequences are given in the SI. It should be noted that the attainable SNRs depend on the probe geometry. As detailed in the Supporting Information, the probe sensitivity is readily calculated from the RF pulse duration and power, using the principle of reciprocity.^36,37^ A probe optimized for proton detection will yield a 4-fold better SNR with INEPT, compared to a probe optimized for carbon detection with direct carbon detection. The Supporting Information also shows that, for the same polarization, the sensitivity of detection may be boosted by a factor ≈8 by employing a cryoprobe with detection at a higher magnetic field. The injection device presented here has an outer diameter of only 25 mm and is thus compatible with any 5 mm liquid-state cryoprobe.

**Figure 6:**
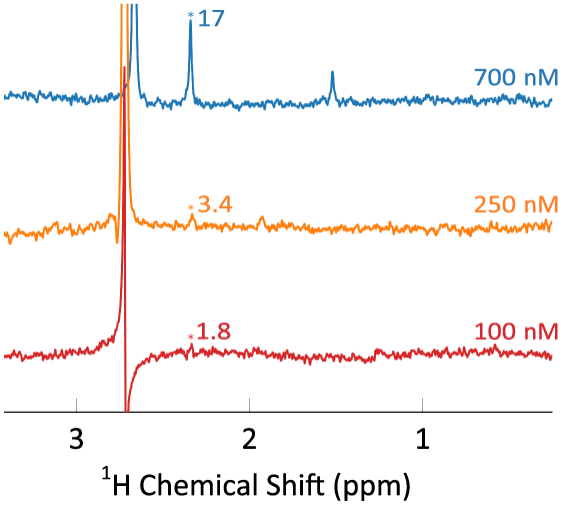
^1^H spectra of 2-^13^C-pyruvate following ^13^C hyperpolarization, rapid transfer, dissolution, and polarization transfer to protons via INEPT. The applied linebroadening is 2 Hz. In the first experiment (top), a 5 μL bead of 100 μM pyruvate was hyperpolarized and dissolved in 700 μL of buffer, yielding a final concentration of 700 nM. The obtained SNR is indicated near the pyruvate peak. In the second experiment (middle), a 1.75 μL bead was used in the same way, yielding a final concentration of 250 nM. For the third experiment (bottom), a 1.5 μL bead of 50 μM was used in the same way, yielding a final concentration of 100 nM.

The resulting ^1^H spectra of three different experiments with different amounts of pyruvate are shown in Figure 6. The signal due to the pyruvate methyl protons appears at 2.3 ppm. The antiphase signal near 2.6 ppm is due to DMSO. At the reported concentration, it is not possible, to measure the thermal equilibrium signal. The SNR, indicated near the respective peaks in Figure 6, is not strictly proportional to the pyruvate concentration, an effect that we attribute to a superior shim for the experiment with a resulting pyruvate concentration of 700 nM. At a concentration of 250 nM, the obtained SNR of 3.4 is close to the limit of detection (SNR = 3).^4^ A third experiment was conducted using a sample mass of 8 ng, resulting in a final pyruvate concentration of 100 nM. A small peak is visible in the resulting spectrum, however the calculated SNR of this peek is below 3. Equivalent experiments have been performed with a BBO probe leading to signal-to-noise ratios which are approximately a factor of 2 smaller than for the BBI probe, as expected from the analysis in the SM. The corresponding data are shown in the SM.

**Figure 7:**
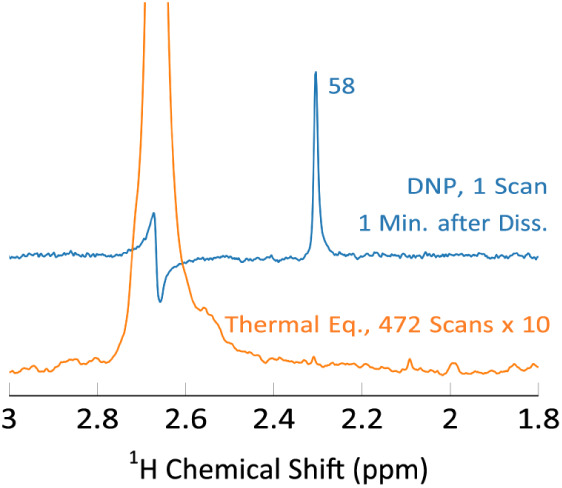
^1^H spectra of 2-^13^C-pyruvate following ^13^C hyperpolarization, rapid transfer, dissolution, a delay of 1 min, and polarization transfer to protons via INEPT. The applied line broadening is 1 Hz. A 4.5 μL bead of 2-^13^C-pyruvate (5 mM) was hyperpolarized and dissolved in 700 μL of buffer, yielding a final concentration of 45 μM. As detailed in the SM, the ^1^H enhancement is estimated to be 350, indicating a ^13^C-enhancement, 1 min after dissolution, of ∼4000. With the measured ^13^C T1 value of 24 s, this corresponds to a ^13^C polarization immediately after dissolution of 40%.

In experiments in clinical settings, where metabolic conversion of pyruvate is monitored, it is not the sensitivity directly after dissolution, which is most relevant, but the sensitivity after e.g., a minute during which the conversion has proceeded. To determine the SNR obtainable with our approach after a period of 1 min, the bullet-DNP experiment was performed with a final pyruvate concentration of 45 μM, using the BBO probe. The resulting ^1^H spectrum has an SNR of 58 (Figure 7). In a proton-polarized experiment, essentially no hyperpolarization would be left after this long period, and in a carbon-polarized, carbon observed experiment the SNR would be 4–8 times lower, depending on the probe. Thus, the carbon-polarized, proton-detected approach seems to be superior for this type of experiments.

The high concentration used in the latter experiment allows us to estimate that the ^1^H signal enhancement, 1 min after dissolution, is approximately 350. This corresponds to a ^13^C enhancement of 4000. At the measured ^13^C *T*_1_ value of 24 s, this corresponds to an initial carbon polarization of 40%, in reasonable agreement with the measurement of the solid-state polarization (34%). Assuming the same initial carbon polarization, the SNR for an experiment with a final concentration of 420 nM and immediate detection, shown in the SM, can be estimated as 58(0.42*/*45)*/*12 ≈ 6, where the factor 1/12 arises from the *T*_1_ losses in the experiment due to the 1 min delay. This value is in good agreement with the experimentally observed SNR of 4.6.

The INEPT strategy that we have used has previously been found to be ineffective for imaging applications of hyperpolarized 1-^13^C lactate at high field.^16^ This is because the reported fast *T*_2_ relaxation (100 ms for protons at 7 T) in *in vivo* MRI applications relaxes a substantial fraction of the magnetization before the INEPT transfer is complete. However, in 2-^13^C-pyruvate the *J*-coupling (7 Hz) between the methyl protons and the ^13^C nucleus is almost two times stronger than the 1-^13^C-Methyl *J*-coupling (4.1 Hz). In the absence of relaxation, the maximum polarization is transferred already after 70 ms of proton transverse evolution, and 80% of the maximum are transferred within only 40 ms. This time is (substantially) shorter than the *in vivo T*_2_ at high-field, indicating that 2-^13^C-pyruvate is a more suitable precursor for reverse INEPT in hyperpolarized imaging experiments. As shown in the SM, with a *T*_2_ of 100 ms, the fraction of ^1^H magnetization that can be obtained is 55% of the theoretical estimate in the absence of relaxation. Therefore, 2-^13^C pyruvate is a more compelling candidate for the application of reverse INEPT to hyperpolarized in vivo experiments.

## Extension to a Mixture of Amino Acids

In order to explore the applicability of INEPT for the analysis of mixtures, we prepared a solution containing 1-^13^C Gly (1.3 mM), 6-^13^C Ile (1.2 mM), 6-^13^C Leu (1.7 mM), and 1-^13^C Ala (1.1 mM) in D_2_O/DMSO (2:1). The narrow-band radical trityl was again included at a concentration of 15 mM, and carbon spin diffusion was again facilitated by the addition of 1 M doubly labeled DMSO ^13^C. We used a volume of 10 μL of this solution in the same way we described previously for pyruvate. A second layer of 10 μL D_2_O/DMSO (2:1) was added on top to seal the sample and limit sample heating during transfer. Care was taken to prevent mixing of the sample and the final layer.

The bullet was hyperpolarized for approximately 6 h and subsequently shot into an injection device inside the 9.4 T liquid-state NMR magnet as described earlier. The volume of liquid used for dissolution was 700 μL, resulting in concentrations of the amino acids of approximately 20 μM.

A ^13^C spectrum and a ^1^H spectrum were recorded sequentially on a single hyperpolarized sample. A 15° flip angle pulse was used for ^13^C excitation. Following ^13^C signal acquisiton, an INEPT transfer was carried out, and the proton signal was observed. The spin systems of the four amino acids have differing *J* couplings and proton multiplicities. To achieve an efficient polarization transfer, the delays (see SM) were set to DEL1 = 1/6 *J* and DEL2 = 1/4 *J* with *J* = 7 Hz. The experiment was carried out using the BBO probe.

**Figure 8:**
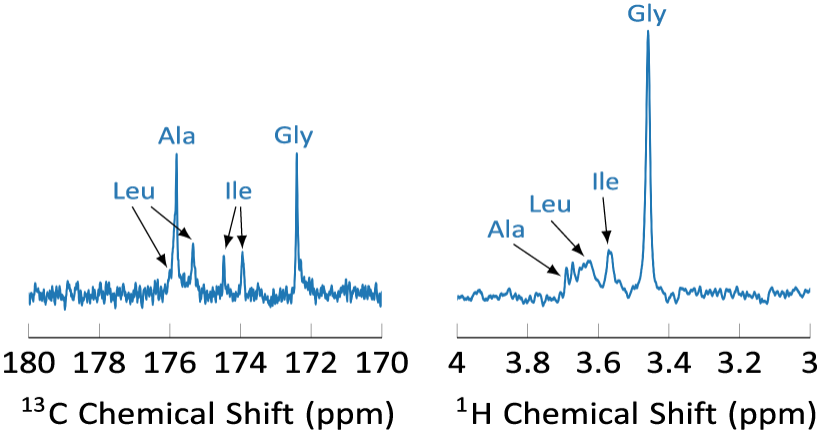
^13^C (left) and ^1^H (right) spectra of a hyperpolarized amino acid mixture. The amino acid concentrations after dissolution are Gly (19 μM), Ile (17 μM), Leu (24 μM), and Ala (16 μM). The ^13^C signal assigment is Gly (172.4 ppm), Ile (doublet, 174.47, 174.97), Leu (doublet, 175.88, 175.35), and Ala (175.82 ppm). The observed doublets are due to coupling with neighboring carbons. One of the Leu doublet peaks is masked by the Ala peak. The ^1^H INEPT spectrum comprises a singlet due to the two Gly-C_α_ protons (3.44 ppm), a doublet due to the Ile-C_α_ proton (3.55 ppm), a triplet due to the Leu-C_α_ proton (3.61 ppm), and a quartet due to the Ala-C_α_ proton (3.66 ppm).

The resulting spectra are shown in Figure 8. The amino acids are readily detected both in the proton and the carbon spectrum at low micromolar concentrations. The signal intensities are in the expected range for the ^13^C spectrum (where carbon–carbon couplings have to be taken into account) as well as in the ^1^H spectrum (where proton–proton couplings and the number of protons have to be taken into account).

## Conclusion and Outlook

We have shown that carbon hyperpolarization, followed by a transfer of magnetization from carbon to protons yields superior mass sensitivity, even when the final concentrations are in the nanomolar range. The detection is made possible through improvements to the bullet-DNP instrument, an optimal choice of the bullet material, and an optimized substrate for the direct polarization of ^13^C labeled moieties at low concentrations.

The sensitivity of the experiments reported here can be boosted by approximately 1 order of magnitude by employing a cryoprobe and detection at a higher magnetic field,^38^ with a resulting limit of detection of 30 nM. The higher sensitivity of such a scheme could also be put to use to implement the experiments described here at natural abundance, which correspondingly would be observable at concentrations down to 3 μM. The mass sensitivity can be boosted by up to 2 orders of magnitude if the solvent volume is reduced and if the sample is then detected using a miniaturized detector.^2,4,20,39^

For applications of INEPT to hyperpolarized MRI, fast *T*_2_ relaxation may reduce the attainable ^1^H polarization, in particular at high field. Then, 2-^13^C-pyruvate with its stronger *J*-coupling to the methyl protons exhibits a fast polarization transfer, making it a candidate for in vivo detection with optimal sensitivity. The sensitivity gains shown in this manuscript would then prolong the time span over which metabolism can be monitored in hyperpolarized MRI experiments.

The methodology presented here can also be applied to the analysis of mixtures, where it can give simultaneous access to ^1^H and ^13^C spectral information at low micromolar concentrations with high mass sensitivity. Work toward unsupervised, serial DNP experiments that improve the throughput and repeatability of such experiments is under way in our lab.

## Data Availability

The original data used in this publication are made available in a curated data archive at KIT open under the DOI: https://doi.org/10.35097/aeiBlRjlEYdPYTaq.

## Supporting Information Available

*T*_1_ fit results, complementary DNP experiments, INEPT simulation, NMR probe sensitivity (PDF)
