# Supplementary material for: Bullet-DNP Enables NMR Spectroscopy of Pyruvate and Amino Acids at Nanomolar to Low Micromolar Concentrations
Source: Anal Chem. 2024 Sep 3;96(37):14734–40. doi: 10.1021/acs.analchem.4c00618 (PMC11411493; doi:10.1021/acs.analchem.4c00618)
Supplement: Supplementary file 1 — ac4c00618_si_001.pdf [file ac4c00618_si_001.pdf]

Supplementary information for *Bullet-DNP enables NMR Spectroscopy of Pyruvate and Amino Acids at Nanomolar to Low Micromolar Concentrations*

Pooja Narwal,[1] Nils Lorz,[2] Masoud Minaei,[1] Sami Jannin,[3] Karel Kouřil,[1] Alvar Gossert,[2,*] and Benno Meier[1,4,†]

[1]*Institute of Biological Interfaces 4, Karlsruhe Institute of Technology, 76344 Eggenstein-Leopoldshafen, Germany*
[2]*Department of Biology, ETH Zürich, 8093 Zürich, Switzerland*
[3]*Université Claude Bernard Lyon 1, CRMN UMR-5082, CNRS, ENS Lyon, Villeurbanne 69100, France*
[4]*Institute of Physical Chemistry, Karlsruhe Institute of Technology, 76131 Karlsruhe, Germany*

(Dated: May 30, 2024)

CONTENTS

I. T_1 Fit Results — S1

II. Reverse INEPT Pulse Sequence — S2
A. Sequence — S2
B. Simulation — S2
C. Simulation with short T_2 — S3

III. Sensitivity gain of INEPT for different probes — S3

IV. Determination of SNR values — S4

V. Cryoprobe sensitivity gain — S5

VI. Repetition of the DNP buildup experiments and spectra from DNP buildups — S5

References — S6

I. T_1 FIT RESULTS

Nucleus	R_0 (s^{-1})	m (s^{-1}/mmol)	T_1^0 (s)
^1H	1.106	0.129	0.904
$^{13}C_1$	0.062	0.017	16.205
$^{13}C_2$	0.042	0.014	23.828
$^{13}C_3$	0.392	0.018	2.550

TABLE S1. Fit Results from T_1 measurements of pyruvate for various concentrations of OX063

The dependence of pyruvate ^{1}H and ^{13}C T_1 on the concentration of trityl OX063 in aqueous solution was measured at a field of 9.4 Tesla using an inversion recovery sequence. The data are shown in the manuscript, here we give the results of the linear fits to the relaxation data.

The relaxation rate for a given concentration can be calculated as

$$R(c) = R_0 + m \times c \tag{S1}$$

where c is the trityl concentration (in mmol).

Note that the trityl concentration used for DNP is typically 15 mM. For 10-fold or higher dilution, the relaxivity of trityl is therefore acceptable. This has to be contrasted with nitroxide radicals, which are typically used at concentrations of 50 mM during DNP, and which can dominate relaxation at low millimolar concentrations.(1)

* alvar.gossert@biol.ethz.ch
† benno.meier@kit.edu

II. REVERSE INEPT PULSE SEQUENCE

A. Sequence

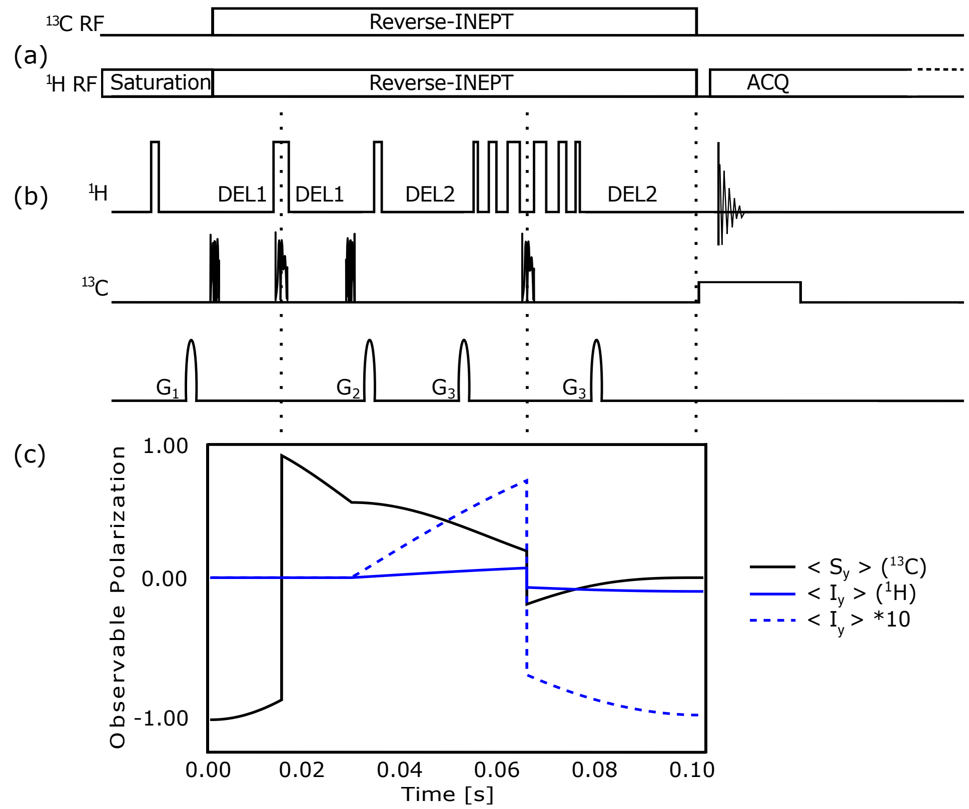

FIG. S1. Reverse INEPT pulse sequence. (a) The INEPT pulse sequence used in this work comprises a saturation block, in which the proton spins are saturated, a reverse INEPT block, in which polarization is transferred from carbon to protons, and an acquisition block. Panel (b) shows the pulse sequence for the reverse-INEPT module shown in panel (a). The broader bars are 180 and the narrow ones represent 90 pulses, respectively. In order to suppress water, the WG3919 pulse is used as a second 180 on ^{1}H. On ^{1}3C channel, the first and second 90 shape pulses are excitation and flip-back pulses. The first 180 shape pulse was the refocussing pulse. Band-selective pulses of the Q5 sebop (350 µs) and Q3 surop (270 µs) kind, both with approximately 8 kHz excitation bandwidth as implemented in the Bruker library, were used for selective excitation and inversion of methyl and carbonyl nuclei. Pulsed-field gradients were applied with the following relative strengths: G1: 61%, G2: 35%, G3: 79%. Phase cycling was not used in this single scan experiment with DNP enhancement. The delays are DEL1 = 1/10 J and DEL2 =1/4 J as appropriate for the refocused INEPT transfer of polarization from the single carbon spin to the three methyl protons (2). The value of J = 6.2 Hz was determined from the respective ^{1}H doublet. Panel (c) shows a SpinDynamica(3) simulation of the expectation values of the transverse magnetization during the reverse INEPT block. The values are normalized to the thermal equilibrium longitudinal magnetization of ^{13}C and ^{1}H, respectively.

B. Simulation

```
In[1]:= $Path=Append[$Path,"/Path/ToSpinDynamica/SDv3.7.1/SpinDynamica"];

In[2]:= Needs["SpinDynamica"`];

In[3]:= Quiet[SetSpinSystem[4]];
        Ispins = {1, 2, 3};
        Sspins = {4};
        rhoeq = (ThermalEquilibriumDensityOperator[LarmorFrequency[1, 9.4] opI[{Ispins}, "z"], 300] +
            ThermalEquilibriumDensityOperator[LarmorFrequency[13, 9.4] opI[{Sspins}, "z"], 300]);
        Meq = OperatorAmplitude[rhoeq -> opI["z"]];
```

```
PLO = PolarizationLevelOperator[Ispins]
P1H = OperatorAmplitude[rhoeq -> PLO] // EngineeringForm
PLO = PolarizationLevelOperator[4];
P13C = OperatorAmplitude[rhoeq -> PLO] // EngineeringForm
```

```
In[4]:= HJ[J_]=2 π 7 opI[1,"z"].opI[4,"z"]+2 π 7 opI[2,"z"].opI[4,"z"]+2 π 7 opI[3,"z"].opI[4,"z"];
HJ[J_]/. {J->7}
Hlab=LarmorFrequency[13,9.4] opI[{4},"z"];
ρΘ=ThermalEquilibriumDensityOperator[Hlab,300];
Meq=OperatorAmplitude[ρΘ->opI[{Sspins},"z"]];
L=CombineGenerators[HJ[J_]];

(*start with only 13C polarization *)
ρeq=ThermalEquilibriumDensityOperator[LarmorFrequency[13,9.4] opI[{Sspins},"z"],300];

INEPT[τ_]:={RotationSuperoperator[{Sspins},{π/2,"x"}],{None,0.4τ},
RotationSuperoperator[{π,"x"}],{None,0.4τ},RotationSuperoperator[{π/2,"y"}],
{None,τ},RotationSuperoperator[{π,"x"}],{None,τ}};

trajy1 =Trajectory[ρeq->opI[1,"y"],INEPT[1/(4 7)], BackgroundGenerator->L,
NormalizationFactor -> 32.0132×10^-6/8];

trajy4= Trajectory[ρeq->opI[4,"y"],INEPT[1/(4 7)], BackgroundGenerator->L,
NormalizationFactor ->8.05144×10^-6/8];

trajy10= Trajectory[ρeq->opI[1,"y"],INEPT[1/(4 7)], BackgroundGenerator->L,
NormalizationFactor ->32.0132×10^-6/8/10];

Plot[{trajy4[t], trajy1[t], trajy10[t]},{t,0,Duration[INEPT[1/(4 7)]]},
FrameLabel->{"Time[s]"},
PlotLegends->{"<Sy> (13C)","<Iy> (1H)","<Iy>* 10"},
PlotStyle->{Black,Blue, {Blue, Dashed}}]
```

C. Simulation with short T_2

III. SENSITIVITY GAIN OF INEPT FOR DIFFERENT PROBES

The data shown in the main manuscript show the signal-to-noise ratio for direct detection of a single ^{13}C, for direct detection of the three methyl ^{1}H, and for detection of ^{13}C via reverse INEPT to the three methyl protons. The attained values are 27, 2900, and 230, respectively. These spectra have been measured using a so-called BBI probe, which has an inner coil for the detection of ^{1}H and an outer coil for the detection of ^{13}C. This geometry is the most sensitive for proton detection. However, for direct ^{13}C detection, one would use a so-called BBO probe, which has an inner coil for ^{13}C detection.

The 400 MHz BBO probe in our lab has a ^{13}C $\pi/2$-pulse duration of 10 µs at a power of 50 W.

The 400 MHz BBI probe used for the sensitivity comparison has a ^{13}C $\pi/2$ pulse duration of 15.8 µs at 75 W.

Since B_1 scales with $\sqrt{P}$ (5), and the pulse-duration is inversely proportional to B_1, the pulse duration of the BBI probe at 50 W would be $15.8/\sqrt{50/75}\mu\text{s} = 19.4\mu\text{s}$, i.e. approximately twice as long as on the BBO probe. For a given RF power level, the B_1 strength is therefore approximately half of that achieved on the BBO probe. By the principle of reciprocity (6), also the voltage induced in the RF coil for a given magnetization is halved, and the ^{13}C signal-to-noise ratio attainable with the BBI probe is half that of the one attainable on a BBO probe. Since the INEPT sensitivity gain on the BBI probe is $230/27 \approx 8.6$, the sensitivity gain of INEPT on a BBI probe, compared to direct detection on a BBO probe is $8.6/2 \approx 4$.

A set of hyperpolarized experiments with the BBO probe is shown in Fig. S3. It can be seen that the attained SNR is approximately two-fold lower than that for the datasets shown in the main manuscript (which were recorded using a TBI probe, with sensitivity equivalent to a BBI probe).

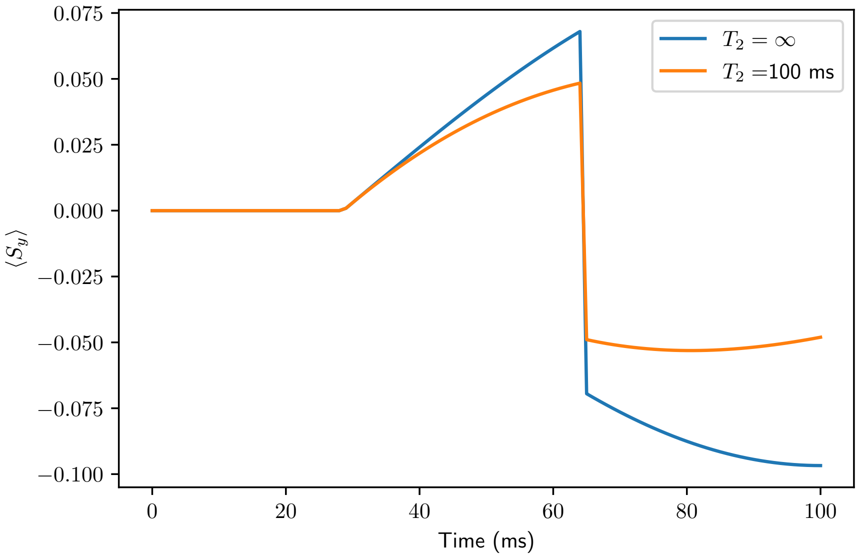

FIG. S2. Simulation without relaxation (blue curve), and with fast T_2 relaxation as it occurs in 7 T *in vivo* MRI experiments.(4) In the latter case, the proton magnetization peaks approximately 50 ms after the π-pulse at a value corresponding to 0.55 times the value that would be obtained without relaxation.

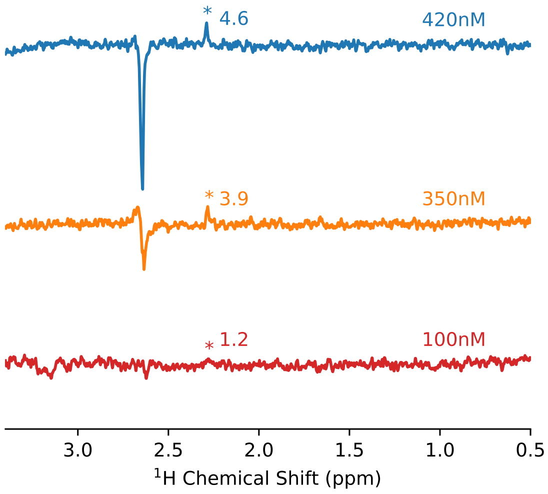

FIG. S3. ^{1}H spectra of hyperpolarized 2-^{13}C-pyruvate. The experimental procedure is exactly like that described in the manuscript, but the data were recorded using a BBO probe and hence have an approximately 2-fold lower SNR for a given concentration. With this probe, pyruvate is detectable at 350 nM, but at 100 nM only a faint indication of the presence of pyruvate can be seen in the spectrum.

IV. DETERMINATION OF SNR VALUES

The signal-to-noise ratios are calculated in pyNMR (7), using the same implemenation as that in TopSpin. For all values reported in the manuscript, the interval [1 ppm, 2 ppm] is chosen as the noise region.

Then a linear regression is performed in this interval, and the resulting straight line is subtracted from the spectrum. This procedure effectively removes any remaining linear baseline in the interval. The corrected spectrum may be written as

$$S_n^c(i) = S_n(i) - (a + bi) \tag{S2}$$

The coefficients a and b are calculated directly from the experimental data. We refer to Reference (8) for details on the linear

regression procedure.

The noise is then calculated as

$$N = \sqrt{\sum_i (S_n^c(i))^2/(n-1)} \tag{S3}$$

where the sum is over the n spectral points in the noise region.

The SNR is then given as

$$\mathrm{SNR} = P/(2N) \tag{S4}$$

where P is the peak is the maximum value of the spectrum in the region form 2.1 to 2.4 ppm, corresponding to the pyruvate signal.

V. CRYOPROBE SENSITIVITY GAIN

In order to estimate the additional sensitivity gain that could be obtained using a cryoprobe, the Bruker standard sucrose sample was measured using the 400 MHz probes used in this work, as well as an optimized CP-TCI 600 MHz cryoprobe at ETH Zürich. The SNRs were obtained using the Bruker macro sup cal. The results are shown in Tab. S2. After correction for the higher polarization due to the higher magnetic field strength (division by 1.5), the SNR obtained with the cryoprobe is 8.5 times larger than the one obtained with the most sensitive probe available for hyperpolarization experiments. With the latter probe, a 250 nm concentration of pyruvate was sufficient to detect pyruvate with an SNR above the limit of detection (3). Correspondingly, a concentration of 250 nm / 8.5 ≈ 30 nm would be sufficient to achieve an SNR > 3 when the hyperpolarization experiments detailed in this work are combined with such a probe.

Probe	^{1}H Frequency	SNR	SNR at 400 MHz polarisation
TBI 400S1 H/C-BB-D-05 Z	400 MHz	102	102
BBO 400S1 BBF-H-D-05 Z	400 MHz	71	71
CP-TCI H & F-N/C-D-05 Z	600 MHz	1295	863

TABLE S2. Sensitivities of different Bruker probes as estimated using the Bruker sucrose standard sample. Pulse sequence: zgpr. Acquisition parameters: TD 1 s, 8 scans, 4 dummy scans, d1 5 s.

VI. REPETITION OF THE DNP BUILDUP EXPERIMENTS AND SPECTRA FROM DNP BUILDUPS

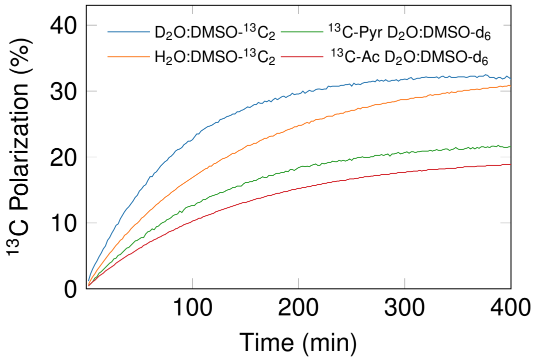

FIG. S4. Rerun of the DNP buildup experiments. for 2-^{13}C pyruvate at 6.7 T with 15mM OX063 trityl radical at a temperature of 1.5 K. The green curve shows a buildup of 1 M 2-^{13}C pyruvate in D_2O:DMSO-d_6 (2:1). The red curve shows a buildup of 84 mM 2-^{13}C pyruvate with 1.6 M 1-^{13}C acetate in D_2O:DMSO-d_6 (2:1). The blue and orange curves show buildups of 100 μM 2-^{13}C pyruvate in D_2O:DMSO-$^{13}C_2$ (2:1) and H_2O:DMSO-$^{13}C_2$ (2:1), respectively. For these two buildups, 1 M of DMSO is substituted with doubly-labelled DMSO-$^{13}C_2$. The polarization is calculated by comparing the signal intensity to a thermal equilibrium signal of a pyruvic acid sample, normalizing for sample volume and molarity for each measurement. The results show only minor deviation with respect to those shown in Fig. 3 of the main manuscript.

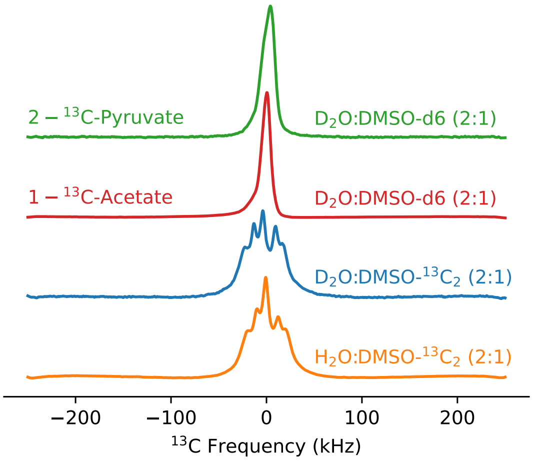

FIG. S5. ^{13}C NMR spectra of the hyperpolarized samples at the end of the DNP buildup process. All spectra were normalized with respect to sample volume and molarity. The DNP buildup spectra were acquired for 2-^{13}C pyruvate at a magnetic field of 6.7 T, employing 15 mM OX063 trityl radical, at a temperature of 1.5 K. Different combinations of solvents were used for these experiments, as indicated on the respective spectrum. ^{13}C-labelled DMSO exhibits a substantially broader resonance than 1-^{13}C-acetate or 2-^{13}C-pruvate. Deuteration of the water fraction of the mixture has only a small influence on the observed lineshape, but a substantial influence on the buildup dynamics (as seen in Fig. S4).

---

(1) P. Miéville, P. Ahuja, R. Sarkar, S. Jannin, P. R. Vasos, S. Gerber-Lemaire, M. Mishkovsky, A. Comment, R. Gruetter, O. Ouari, P. Tordo, and G. Bodenhausen, Angewandte Chemie International Edition **49**, 6182 (2010).

(2) T. Claridge, *High-resolution NMR Techniques in Organic Chemistry*, High-Resolution NMR Techniques in Organic Chemistry No. Bd. 19 (Elsevier Science, 1999).

(3) C. Bengs and M. H. Levitt, Magnetic Resonance in Chemistry **56**, 374 (2017).

(4) J. Wang, F. Kreis, A. J. Wright, R. L. Hesketh, M. H. Levitt, and K. M. Brindle, Magnetic Resonance in Medicine **79**, 741 (2017).

(5) C. P. Slichter, Springer Series in Solid-State Sciences (1990), 10.1007/978-3-662-09441-9.

(6) D. Hoult and R. Richards, Journal of Magnetic Resonance (1969) **24**, 71 (1976).

(7) Benno Meier, pynmr, (2024), available from https://github.com/bennomeier/pynmr.

(8) P. K. Janert, *Data Analysis with Open Source Tools* (O'Reilly, Sebastopol, CA 95472, 2010).
